# Evaluation of chromosomal abnormalities and copy number variations in fetuses with ultrasonic soft markers

**DOI:** 10.1186/s12920-021-00870-w

**Published:** 2021-01-12

**Authors:** Meiying Cai, Na Lin, Xuemei Chen, Meimei Fu, Nan Guo, Liangpu Xu, Hailong Huang

**Affiliations:** Department of the Prenatal Diagnosis Center, Fujian Maternity and Child Health Hospital, Affiliated Hospital of Fujian Medical University, Fujian Key Laboratory for Prenatal Diagnosis and Birth Defect, Fuzhou, China

**Keywords:** Single nucleotide polymorphism array analysis, Ultrasonic soft markers, Copy number variations, Fetus

## Abstract

**Background:**

Some ultrasonic soft markers can be found during ultrasound examination. However, the etiology of the fetuses with ultrasonic soft markers is still unknown. This study aimed to evaluate the genetic etiology and clinical value of chromosomal abnormalities and copy number variations (CNVs) in fetuses with ultrasonic soft markers.

**Methods:**

Among 1131 fetuses, 729 had single ultrasonic soft marker, 322 had two ultrasonic soft markers, and 80 had three or more ultrasonic soft markers. All fetuses underwent conventional karyotyping, followed by single nucleotide polymorphism (SNP) array analysis.

**Results:**

Among 1131 fetuses with ultrasonic soft markers, 46 had chromosomal abnormalities. In addition to the 46 fetuses with chromosomal abnormalities consistent with the results of the karyotyping analysis, the SNP array identified additional 6.1% (69/1131) abnormal CNVs. The rate of abnormal CNVs in fetuses with ultrasonic soft marker, two ultrasonic soft markers, three or more ultrasonic soft markers were 6.2%, 6.2%, and 5.0%, respectively. No significant difference was found in the rate of abnormal CNVs among the groups.

**Conclusions:**

Genetic abnormalities affect obstetrical outcomes. The SNP array can fully complement conventional karyotyping in fetuses with ultrasonic soft markers, improve detection rate of chromosomal abnormalities, and affect pregnancy outcomes.

## Background

With the rapid development of high-resolution ultrasound and prenatal ultrasound diagnostic techniques, some ultrasonic soft markers can be found during ultrasound examination [1]. These ultrasonic soft markers include thickened nuchal translucency, ventriculomegaly, absent nasal bone, hyperechogenic bowel, choroid plexus cyst, short femur, echogenic intracardiac focus, mild tricuspid regurgitation, pyelectasis, single umbilical artery, etc. In recent years, studies found an increased risk of chromosomal abnormalities in fetuses with ultrasonic soft markers. However, ultrasonic soft markers mainly refer to a non-specific index, which does not completely indicate the structural abnormality of the fetus, and may be a normal variation [2]. However, whether the fetus with ultrasonic soft markers should be diagnosed by prenatal diagnosis is still debated.

To explore the relationship among ultrasonic soft markers, genetic abnormalities in fetus, and its influence on pregnancy outcome, we systematically analyzed the relationship between ultrasonic soft markers and genetic abnormalities by observing 1131 fetuses with ultrasonic soft markers, aiming at expounding the utility of SNP array in fetus with ultrasonic soft markers.

## Methods

### Retrospective samples

From November 2016 to July 2019, 1131 fetuses with ultrasonic soft markers underwent prenatal examination with consent after proper counseling at the Prenatal Diagnosis Center of the Fujian Provincial Maternal and Children Health Hospital. The mean maternal age was 28.9 (18–47) years, and the mean gestational week was 24.3 (13–38) weeks. Ultrasonic soft markers were diagnosed according to Li Shengli’s diagnostic criteria [3]. The inclusion criteria for ultrasonic soft markers were as follows: thickened nuchal translucency, ventriculomegaly, absent nasal bone, hyperechogenic bowel, choroid plexus cyst, right subclavian vagus, short femur, echogenic intracardiac focus, mild tricuspid regurgitation, pyelectasis, single umbilical artery, and alteration of wave A in the ductus venosus. The exclusion criteria were ultrasonic structural malformation and gemellary pregnancy. According to the number of ultrasonic soft markers, the fetuses were divided into a single ultrasonic soft marker group, two ultrasonic soft markers group, and three or more ultrasonic soft markers group.

Fetal samples were collected by chorionic villus sampling (n = 20), amniocentesis (n = 783), and cord blood sampling (n = 328) according to the gestational age. The villus was collected by chorionic villus sampling at 13–15 gestational weeks, amniotic fluid was collected by amniocentesis at 16–24 gestational weeks, and umbilical cord blood was collected by cordocentesis after the 24th gestational week.

The study was approved by the Fujian Provincial Maternal and Child Health Hospital regarding ethical conduct of research.

### Conventional karyotyping

Fetal sample cells were cultured and analyzed by conventional karyotyping with Giemsa banding at a resolution of 450–550 bands.

### SNP array

Digestion, amplification, purification, fragmentation, labeling, hybridization with chips, washing, scanning, and data analysis of sample genomic DNA were carried out according to the operation manual provided by Affymetrix Company of the United States. Chromosome Analysis Suite software 3.3 (Affymetrix, Santa Clara, CA, USA). and annotated based on genome version GRCh37 (hg19) were used to analyze the results. The referenced databases included the Database of Genomic Variants (DGV), Database of genomic variation and Phenotype in Humans using Ensembl Resources (DECIPHER), Online Mendelian Inheritance in Man (OMIM), International Standards for Cytogenomic Arrays (ISCA), and Global Affymetrix User Pathology Shared Database (CGDB). According to the American College of Medical Genetics (ACMG) guidelines [4], CNVs were classified as being pathogenic, benign, or variations of uncertain clinical significance (VUS). All abnormal CNVs were verified by fluorescence in situ hybridization. Parental testing was carried out for fetuses who had abnormal CNVs to determine inheritance pattern. The microarray data from this study were submitted to the Gene Expression Omnibus repository (https://www.ncbi.nlm.nih.gov/geo/query/acc.cgi?acc=GSE163799).

### Statistical analyses

Statistical analyses were performed using SPSS Statistics 20 software (IBM Corp., Armonk, NY). *P* value of < 0.05 indicated statistical significance. Chi-square test was used to analyze the detection rate of chromosomal abnormalities and abnormal CNVs among the ultrasonic soft markers groups.

## Results

### Fetal profile

There were 1131 fetuses with ultrasonic soft markers, including 729 fetuses with a single ultrasonic soft marker, 322 fetuses with two ultrasonic soft markers, and 80 fetuses with three or more ultrasonic soft markers. In 729 fetuses with a single ultrasonic soft marker, 302 had thickened nuchal translucency, 115 had ventriculomegaly, 76 had absent nasal bone, 65 fetuses had hyperechogenic bowel, 54 had choroid plexus cyst, 32 had right subclavian vagus, 26 had short femur, 22 had echogenic intracardiac focus, 16 had mild tricuspid regurgitation, 8 had pyelectasis, 7 had single umbilical artery, and 6 had alteration of wave A in the ductus venosus (Table 1).

**Table 1:** Phenotypic characteristics of 1131 fetuses with ultrasonic soft markers

Classification of fetuses with ultrasonic soft markers	Number of fetuses
Single ultrasonic soft marker	729
Thickened nuchal translucency	302
Ventriculomegaly	115
Absent nasal bone	76
Hyperechogenic bowel	65
Choroid plexus cyst	54
Right subclavian vagus	32
Short femur	26
Echogenic intracardiac focus	22
Mild tricuspid regurgitation	16
Pyelectasis	8
Single umbilical artery	7
Alteration of wave A in the ductus venosus	6
Two ultrasonic soft markers	322
Three or more ultrasonic soft markers	80

### Conventional karyotyping

Conventional karyotyping showed 46 fetuses with chromosome abnormalities, including 35 aneuploid, two mosaic chromosomal number abnormalities, and nine chromosomal structural abnormalities. Moreover, 35 fetuses had aneuploidy, including 23 fetuses with trisomy 21, five fetuses with trisomy 18, three fetuses with trisomy 13, two fetuses with 47, XXY, and two fetuses with 45, X. In nine fetuses, the chromosomal structural abnormalities were as follows: 46,XX,del(5)(5p15.3), 46,XX,del(18)(p11.2), 46,XX,add(5)(p15.3), 46,XY,add(4)(q35), 46,XX,add(X)(q21), 46,XX,add(10)(q26), 46,XY,add(2)(q37), 46,XX,add(12)(p13.3), and 46,XX,del(1)(q21).

Among the 46 fetuses with chromosomal abnormalities, 26 had single ultrasonic soft marker, 13 had two ultrasonic soft markers, and seven had three or more ultrasonic soft markers. The rates of chromosomal abnormalities in fetuses with single ultrasonic soft marker, two ultrasonic soft markers, and three or more ultrasonic soft markers were 3.6% (26/729), 4.0% (13/322), and 8.8% (7/80), respectively (Table 2). No significant difference was found in the rate of chromosomal abnormalities among the ultrasonic soft marker groups (X^2^ = 4.965, *P* = 0.084).

**Table 2:** Ultrasonic soft markers and conventional karyotyping

Groups	Number of fetuses	Rate of chromosomal abnormalities (%)	Trisomy 21	Trisomy 18	Trisomy 13	47,XXY	45,X	Mosaic chromosomal number abnormalities	Chromosomal structural abnormalities
Single ultrasonic soft marker	729	26 (3.6)	12	3	3	1	2		5
Short femur	26	2 (7.7%)							2
Thickened nuchal translucency	302	18 (6.0%)	10	1	3		1		3
Choroid plexus cyst	54	2 (3.7%)		2					
Absent nasal bone	76	2 (2.6%)	1			1			
Ventriculomegaly	115	2 (1.7%)	1				1		
Two ultrasonic soft markers	322	13 (4.0%)	7	1		1		1	3
Three or more ultrasonic soft markers	80	7 (8.8%)	4	1				1	1

### SNP array results

In addition to the 46 fetuses with chromosomal abnormalities consistent with the results of the conventional karyotyping, the SNP array identified additional 6.1% (69/1131) abnormal copy number variations (CNVs). Of the 69 abnormal CNVs, 37 were pathogenic CNVs and 32 were VUS. The rate of abnormal CNVs in fetuses with single ultrasonic soft marker, two ultrasonic soft markers, three or more ultrasonic soft markers were 6.2% (45/729), 6.2% (20/322), and 5.0% (4/80), respectively. No significant difference was found in the rate of abnormal CNVs among the fetuses with ultrasonic soft markers (X^2^ = 0.183, *P* = 0.913) (Table 3). Abnormal CNVs were between 0.2 and 5.6 MB in size. Among the 37 pathogenic CNVs, nine were known microdeletion/microduplication syndrome, namely, four cases of 22q11.2 microdeletion syndrome, three cases of 22q11.2 microduplication syndrome, one case of 3q29 microdeletion syndrome, and one case of 7q11 microdeletion syndrome (Williams-Beuren syndrome). Thirty-two fetuses had VUS CNVs, including 21 fetuses with duplications, seven fetuses with deletions, and four fetuses lacked heterozygosity. VUS CNVs were associated with duplications of 16p13.11, 15q13.3, 2p16.1, 19q13.42, Xq23, 10q21.1, 2q36.1q36.2, 16p13.13p13.12, 8p23.2, 20q13.2, 2p22.3, 10q24.31q24.32, 13q14.3, 6q21; deletions of 15q11.2, 3p22.1, 9q31.3, 3p26.31, and 14q21.2q21.3 (Table 4).

**Table 3:** Ultrasonic soft markers and SNP array

Groups	Number of fetuses	Number of abnormal CNVs	Pathogenic CNVs	VUS CNVs
Single ultrasonic soft marker	729	45 (6.2%)	24	21
Short femur	26	4 (15.4%)	3	1
Mild tricuspid regurgitation	16	2 (12.5%)	1	1
Choroid plexus cyst	54	4 (7.4%)	1	3
Ventriculomegaly	115	8 (7.0%)	4	4
Absent nasal bone	76	5 (6.6%)	2	3
Thickened nuchal translucency	302	18 (6.0%)	11	7
Hyperechogenic bowel	65	3 (4.6%)	2	1
Echogenic intracardiac focus	22	1 (4.5%)	1	0
Two ultrasonic soft markers	322	20 (6.2%)	10	10
Three or more ultrasonic soft markers	80	4 (5.0%)	3	1

*CNVs* abnormal copy number variations, *SNP* single nucleotide polymorphism, *VUS* variation of uncertain clinical significance

**Table 4:** Abnormal CNVs in fetuses with ultrasonic soft marker

Case	Indication	SNP array	Interpretation	Size Mb	Outcome	Inheritance
1	Short femur	arr[hg19]17q21.31(41,774,473–42,491,805) × 4	P	0.7	TP	Denovo
2	Short femur	arr[hg19] 10q21.3(68,972,662–69,925,900) × 1	P	0.9	TP	Denovo
3	Short femur	arr[hg19]4q28.3q31.3(133,718,289–154,569,367)hmz	VUS	20.8	TD	–
4	Short femur	arr[hg19] 15q13.3(32,003,537–32,444,043) × 3	VUS	0.4	TP	Denovo
5	Thickened nuchal translucency	arr[hg19]22q11.21(18,648,855–21,459,713) × 3	P	2.8	TP	Maternal
6	Thickened nuchal translucency	arr[hg19]Yq11.222q11.223(20,252,055–25,863,576) × 0	P	5.6	TP	Denovo
7	Thickened nuchal translucency	arr[hg19] 22q11.21(18,648,855–21,800,471) × 3	p	3.1	TP	Denovo
8	Thickened nuchal translucency	arr[hg19] 3q29(195,678,474–197,340,833) × 1	p	1.6	TP	Denovo
9	Thickened nuchal translucency	arr[hg19] 1p31.3(61,886,890–63,701,576) × 1	P	1.8	TP	Denovo
10	Thickened nuchal translucency	arr[hg19] 22q11.21(20,730,143–21,800,471) × 3	P	1.0	TP	Denovo
11	Thickened nuchal translucency	arr[hg19]1q21.1q21.2(145,829,473–148,520,164) × 1	P	2.7	TP	Denovo
12	Thickened nuchal translucency	arr[hg19]22q11.21(20,716,876–21,800,471) × 1	p	1.0	TP	Denovo
13	Thickened nuchal translucency	arr[hg19] 16p13.11(14,910,158–16,508,123) × 1	P	1.6	TP	Denovo
14	Thickened nuchal translucency	arr[hg19] Xp22.31(6,449,836–8,141,076) × 0, (XY) × 1	P	1.7	TP	Denovo
15	Thickened nuchal translucency	arr[hg19] Xq28(153,560,562–153,826,362) × 3	P	0.3	TP	Denovo
16	Thickened nuchal translucency	arr[hg19] 2p16.1(55,993,333–60,450,583) × 3	VUS	4.5	TD	–
17	Thickened nuchal translucency	arr[hg19] 19q13.42(53,717,606–54,656,213) × 3	VUS	0.9	TD	Denovo
18	Thickened nuchal translucency	arr[hg19]Xq23(109,823,197–110,252,333) × 3	VUS	0.4	TD	–
19	Thickened nuchal translucency	arr[hg19] 15q13.3(32,003,537–32,444,043) × 3	VUS	0.4	TD	Denovo
20	Thickened nuchal translucency	arr[hg19] 10q21.1(53,477,821–55,138,122) × 3	VUS	1.7	TD	Denovo
21	Thickened nuchal translucency	arr[hg19] 9q31.3(113,177,821–113,544,998) × 1	VUS	0.4	TD	–
22	Thickened nuchal translucency	arr[hg19] 1q21.1q21.2(144,077,593–148,750,533) hmz arr[hg19] 3p21.31p21.1(48,166,782–53,172,233) hmz arr[hg19] 5q21.3q22.1(107,196,975–110,478,806) hmz arr[hg19] 12q21.31q21.33(82,446,525–91,707,400) hmz arr[hg19] 14q31.2q32.12(84,339,970–92,755,472) hmz arr[hg19] 15q24.1q25.3(73,065,223–87,467,262) hmz arr[hg19] 16p13.3(94,807–3,112,982) hmz arr[hg19] 17p12p11.2(15,838,698–22,170,994) hmz	VUS	99	TD	–
23	Ventriculomegaly	arr[hg19]16p11.2(29,567,296–30,190,029) × 1	P	0.6	TP	Denovo
24	Ventriculomegaly	arr[hg19]5q35.2q35.3(175,416,095–177,482,506) × 1	P	2.0	TP	Denovo
25	Ventriculomegaly	arr[hg19]Xq28(152,446,333–153,581,657) × 3, 1p36.33p36.23(849,466–592,172) × 1, 1q44(246,015,892–249,224,684) × 3	P	1.1, 7.7, 3.2	TP	Denovo
26	Ventriculomegaly	arr[hg19]1q21.1(145,375,770–145,770,627) × 1, 9p24.1(4,623,660–5,501,699) × 3	P	0.7, 0.9	TP	Denovo
27	Ventriculomegaly	arr[hg19]3p22.1 (42,875,130–43,309,436) × 1	VUS	0.4	TP	Denovo
28	Ventriculomegaly	arr[hg19]2q36.1q36.2(224,459,152–225,330,583) × 3	VUS	0.9	TD	Denovo
29	Ventriculomegaly	arr[hg19]3p26.3(1,855,754–2,663,625) × 1	VUS	0.8	TD	–
30	Ventriculomegaly	arr[hg19] 16p13.11(15,058,820–16,309,046) × 3	VUS	1.3	TD	–
31	Absent nasal bone	arr[hg19]15q13.2q13.3(30,386,398–32,444,261) × 1	P	2.0	TP	Denovo
32	Absent nasal bone	arr[hg19]16p12.2(21,816,542–22,710,614) × 1	P	1.0	TP	Denovo
33	Absent nasal bone	arr[hg19] 15q13.3(32,021,609–32,444,043) × 3	VUS	0.4	TD	Denovo
34	Absent nasal bone	arr[hg19] 15q11.2(22,770,421–23,276,833) × 1	VUS	0.5	TD	Paternal
35	Absent nasal bone	arr[hg19] 16p13.13p13.12(11,528,493–12,934,811) × 3	VUS	1.4	TD	Maternal
36	Hyperechogenic bowel	arr[hg19]22q13.33(49,683,904–51,197,766) × 1	P	3.1	TP	Denovo
37	Hyperechogenic bowel	arr[hg19]10q11.21q11.22(42,433,738–48,006,310) × 1	P	5.5	TP	Denovo
38	Hyperechogenic bowel	arr[hg19]16p13.11(15,171,146–16,309,046) × 3	VUS	1.1	TD	Paternal
39	Choroid plexus cyst	arr[hg19]22q11.21(18,648,855–21,800,471) × 1	P	3.1	TP	Maternal
40	Choroid plexus cyst	arr[hg19] 15q13.3(32,011,458–32,444,043) × 3	VUS	0.4	TD	–
41	Choroid plexus cyst	arr[hg19]8p23.2(3,703,883–5,940,433) × 3	VUS	2.2	TD	–
42	Choroid plexus cyst	arr[hg19] 20q13.2(53,545,723–54,866,110) × 3	VUS	1.3	TD	Denovo
43	Mild tricuspid regurgitation	arr[hg19] 1q21.1(145,287,532–145,966,117) × 3	P	0.7	TP	Paternal
44	Mild tricuspid regurgitation	arr[hg19]16p13.11(15,510,512–16,309,046) × 3	VUS	0.8	TD	–
45	Echogenic intracardiac focus	arr[hg19] 17q12(34,440,088–36,351,919) × 3	P	1.9	TP	Paternal
46	Right subclavian vagus, echogenic intracardiac focus	arr[hg19]22q11.21(18,648,855–21,800,471) × 1	P	3.1	TP	Denovo
47	Cavity of septum pellucidum, amniotic fluid is excessive	arr[hg19] 17p12(14,099,504–15,491,533) × 1	P	1.3	TP	–
48	Echogenic intracardiac focus, pyelectasis	arr[hg19] 6p21.32p21.31(32,965,747–35,234,269) × 3	P	2.2	TP	Denovo
49	Absent nasal bone, echogenic intracardiac focus	arr[hg19] 17p12(14,070,219–15,484,335) × 1	P	1.4	TP	Denovo
50	Absent nasal bone, pyelectasis	arr[hg19] 2p22.3(34,002,379–35,076,738) × 3	VUS	1.0	TD	–
51	Mild tricuspid regurgitation, pericardial effusion	arr[hg19]5p15.33p15.31(4,482,234–6,636,035) × 1	P	2.1	TP	Denovo
52	Short femur, hyperechogenic bowel	arr[hg19]10q11.22q11.23(46,252,072–51,903,756) × 1	P	5.6	TP	–
53	Mild tricuspid regurgitation, hyperechogenic bowel	arr[hg19]15q11.2(22,770,421–23,286,423) × 1	VUS	0.5	TD	Paternal
54	Mild tricuspid regurgitation, echogenic intracardiac focus	arr[hg19] 15q11.2(22,770,421–23,277,436) × 1	VUS	0.5	TD	Denovo
55	Mild tricuspid regurgitation, echogenic intracardiac focus	arr[hg19] Xp22.33 or Yp11.32(387,396–629,998 or 337,396–579,998) × 1	P	0.2	TP	Denovo
56	Echogenic intracardiac focus, ventriculomegaly	arr[hg19] 16p13.11(15,422,960–16,508,123) × 1	P	1.0	TP	Denovo
57	Single umbilical artery, mild tricuspid regurgitation	arr[hg19] 1q21.1q21.2(145,084,525–147,885,600) × 1	P	2.8	TP	Paternal
58	Echogenic intracardiac focus, mild tricuspid regurgitation	arr[hg19] Xp22.33 or Yp11.32(168,551–629,999 or 118,551–579,999) × 1	P	0.5	TP	Denovo
59	Hyperechogenic bowel, mild tricuspid regurgitation	arr[hg19] 16p13.11(15,058,820–16,309,046) × 3	VUS	1.25	TD	Denovo
60	Hyperechogenic bowel, enlargement of intestinal canal	arr[hg19] 16p13.11(14,900,042–16,508,123) × 3	VUS	1.6	TD	Denovo
61	Ventriculomegaly, choroid plexus cyst	arr[hg19]14q21.2q21.3(46,782,405–49,288,860) × 1	VUS	2.5	TP	–
62	Ventriculomegaly, short femur	arr[hg19]3q26.1q29(163,256,369–197,791,601)hmz arr[hg19]5p13.1p11(41,029,137–46,313,469)hmz arr[hg19]6q24.2q25(143,341,406–161,527,784)hmz arr[hg19]12q13.2q21.2(56,011,100–77,134,151)hmz arr[hg19]17q21.2q21.32(39,639,602–45,479,706)hmz arr[hg19]21q21.3q22.2(28,124,165–42,352,287)hmz	VUS	99.1	TD	–
63	Ventriculomegaly, echogenic intracardiac focus	arr[hg19]10q24.31q24.32(102,972,457–103,179,063) × 3	VUS	0.2	TD	Denovo
64	Mild tricuspid regurgitation, pyelectasis	arr[hg19]13q14.3(52,649,105–53,172,866) × 3	VUS	0.5	TD	–
65	Thickened nuchal translucency, thickening of skin in chest and abdomen	arr[hg19]1p33p32.3(50,051,514–53,274,566) hmz arr[hg19]2q23.3q24.1(153,771,280–158,783,675) hmz arr[hg19]3q21.2q22.1(124,817,983–129,317,745) hmz arr[hg19]3q22.1q23(133,262,566–139,418,898) hmz arr[hg19]3q26.1q26.2(161,540,639–168,592,236) hmz arr[hg19]7p22.3p21.2(2,707,569–13,857,235) hmz arr[hg19]8q23.3q24.12(114,788,423–119,897,611) hmz arr[hg19]9p22.1p13.3(19,696,747–36,125,149) hmz arr[hg19]11q12.2q12.3(60,193,879–63,210,491) hmz arr[hg19]11p11.2p11.21(45,781,075–51,550,787) hmz arr[hg19]17q21.31q21.32(41,647,165–44,927,874) hmz arr[hg19]17q25.1q25.3(71,965,953–75,785,426) hmz arr[hg19]20p11.23p11.21(20,268,153–23,275,237) hmz	VUS	77.5	–	–
66	Thickened nuchal translucency, echogenic intracardiac focus, mild tricuspid regurgitation, hyperechogenic bowel	arr[hg19] 18q21.33q22.1(60,147,532–65,974,912) × 1 hmz	P	5.8	TP	–
67	Echogenic intracardiac focus, mild tricuspid regurgitation, pyelectasis	arr[hg19] 6q21(106,385,070–109,072,236) × 3	VUS	2.6	TD	Denovo
68	Ventriculomegaly, hyperechogenic bowel, echogenic intracardiac focus	arr[hg19]16p11.2(28,810,324–29,032,280) × 1	P	0.2	TP	–
69	Echogenic intracardiac focus, mild tricuspid regurgitation, short femur	arr[hg19] 22q11.21(21,059,669–21,800,471) × 1	P	0.4	TP	Denovo

*CNVs* abnormal copy number variations, *P* pathogenic, *TD* term delivery, *TP* termination of pregnancy, *VUS* variation of uncertain clinical significance

The rates of chromosomal abnormalities and abnormal CNVs in fetuses with short femur (23.1%, 6/26) were high, followed by those with mild tricuspid regurgitation (12.5%, 2/16), thickened nuchal translucency (12.0%, 36/302), choroid plexus cyst (11.1%, 6/54), absent nasal bone (9.2%, 7/76), ventriculomegaly (8.7%, 10/115), hyperechogenic bowel (4.6%, 3/65), and echogenic intracardiac focus (4.5%, 1/22). No chromosomal abnormalities and abnormal CNVs of the right subclavian vagus, pyelectasis, single umbilical artery, and alteration of wave A in the ductus venosus were detected.

### Inheritance analysis and pregnancy outcome

We screened inheritance information from nine cases of abnormal karyotype results (exempt 35 cases of aneuploid and two cases of mosaic chromosomal number abnormalities) and 50 cases of abnormal CNVs (exempt the parents refused genetic testing in 19 cases). Inheritance analysis showed that nine fetuses with abnormal CNVs were inherited from unaffected parents, and 50 (nine fetuses with abnormal karyotype results and 40 fetuses with abnormal CNVs) were de novo CNVs. Among 1131 fetuses with ultrasonic soft markers, 1108 fetuses were successfully followed, except for 23 fetuses who were loss to follow-up. Pregnancies of 46 fetuses with chromosomal abnormalities, 37 fetuses with pathogenic CNVs, and three fetuses with VUS CNVs were terminated. Parents of the 29 fetuses with VUS CNVs chose to continue pregnancy, and newborns were followed after birth. Among 29 fetuses with VUS CNVs, one died of unknown cause after birth, two had delayed development after birth, one was lost follow-up, and 25 fetuses were normal development and growth.

## Discussion

In this study, 1131 cases with ultrasonic soft markers were observed as indication for prenatal diagnosis, including 729 fetuses with a single ultrasonic soft marker, 322 fetuses with two ultrasonic soft markers, and 80 fetuses with three or more ultrasonic soft markers. These 1131 fetuses with ultrasonic soft markers were analyzed by conventional karyotyping, and the detection rate of conventional karyotyping was 4.1%. In addition to the 46 fetuses with chromosomal abnormalities consistent with the results of the conventional karyotyping, the SNP array identified additional 6.1% abnormal CNVs.

Trisomy 21 and chromosomal structural abnormalities were the most common chromosomal abnormalities in fetuses with ultrasonic soft markers, accounting for 50% (23/46) and 20% (9/46), followed by trisomy 18, trisomy 13, and other chromosomal number abnormalities. The rate of chromosomal abnormalities in fetuses with single ultrasonic soft marker, two ultrasonic soft markers, and three or more ultrasonic soft markers were 3.6%, 4.0%, and 8.8%, respectively. No significant difference was found in the detection rate of chromosomal abnormalities among fetuses with ultrasonic soft markers. This is obviously different from previous reports that clusters of ultrasonic soft markers greatly increased the likelihood of chromosomal abnormalities compared with a single ultrasonic soft marker [5, 6]. This difference may be related to the selected cohort of fetuses with ultrasonic soft markers.

Among 1131 fetuses with ultrasonic soft markers, in addition to the 46 fetuses with chromosomal abnormalities consistent with the results of karyotyping analysis, the SNP array also identified abnormal CNVs, and no significant difference was found in the detection rate of abnormal CNVs among fetuses with ultrasonic soft markers. Previous studies [7–10] have reported that SNP array was used in fetuses with ultrasonic structural abnormalities, and there are few reports in which SNP array is used in fetuses with ultrasonic soft markers. We used SNP array to detect abnormal CNVs in fetuses with ultrasonic soft markers, and the SNP array results showed that the detection rate of abnormal CNVs was slightly higher than the previous literature [11]. Therefore, SNP array is also recommended to examine fetuses with ultrasonic soft markers.

The rates of chromosomal abnormalities and abnormal CNVs in fetuses with short femur, thickened nuchal translucency, choroid plexus cyst, absent nasal bone, and ventriculomegaly are higher than those with other ultrasonic soft markers. Prenatal diagnosis is necessary for these fetuses, as reported in previous studies [12–14]. Chromosomal abnormalities were not found in fetuses with mild tricuspid regurgitation, hyperechogenic bowel, and echogenic intracardiac focus, but those of fetuses with abnormal CNVs were 12.5%, 4.6%, and 4.5%, respectively. According to reports [15, 16], mild tricuspid regurgitation occurred in 7% of normal fetuses. In this study, only 16 fetuses had mild tricuspid regurgitation, but two fetuses with mild tricuspid regurgitation had abnormal CNVs. Shuster and Keunen [17] considered abnormal CNVs in fetuses with hyperechogenic bowel. In this study, 65 fetuses with hyperechogenic bowel had normal karyotyping analysis results, but three fetuses with hyperechogenic bowel had abnormal CNVs. At present, many studies reported that fetuses with echogenic intracardiac focus did not have an increased risk for chromosomal abnormalities [18–20], but one pathogenic CNV was detected in 22 fetuses with echogenic intracardiac focus in this study. Therefore, SNP array should be considered when karyotyping analysis is carried out for fetuses with mild tricuspid regurgitation, hyperechogenic bowel, and echogenic intracardiac focus. In addition, no chromosomal abnormalities and abnormal CNVs were found in fetuses with right subclavian vagus, pyelectasis, single umbilical artery, and alteration of wave A in the ductus venosus. The sample size of fetuses with right subclavian vagus, pyelectasis, single umbilical artery, and alteration of wave A in the ductus venosus is relatively small; thus, more cases should be accumulated in the future.

Of the 69 abnormal CNVs, 37 were pathogenic CNVs. Although the clinical manifestations of patients with pathogenic CNVs are relatively mild compared with those with chromosomal aneuploidy, clinical manifestations of pathogenic CNVs cannot be ignored, as they can lead to various other clinical manifestations such as developmental delay, low intelligence, and autism [21]. 22q11.2 microdeletion syndrome, 22q11.2 microduplication syndrome, 3q29 microdeletion syndrome, and Williams-Beuren syndrome can lead to serious conditions such as stunting, mental retardation, and epilepsy, bringing heavy economic and mental burden to families. The application of SNP in prenatal diagnosis is particularly important.

Genetic abnormalities affect pregnancy outcomes. When the genomes of fetuses with ultrasonic soft markers are abnormal, their parents are more likely to terminate the pregnancy. In our study, pregnancies of 46 fetuses with chromosomal abnormalities, 37 with pathogenic CNVs, and three with VUS CNVs were terminated. SNP array can provide more objective theoretical basis for correctly evaluating the prognosis of fetus, and prenatal clinical counseling may help pregnant women decide on whether to continue the pregnancy.

SNP array improves the detection rate of chromosomal abnormalities, but SNP array can detect more VUS CNVs, which is a great challenge to genetic counseling. In this study, the detection rate of VUS CNVs was 2.8%. In many cases, even after comparison of SNP array results of the parents, it is still impossible to accurately interpret the clinical characteristics of VUS. This may cause anxiety to pregnant women and even unnecessary termination of pregnancy [22–24]. Thus, a good working algorithm is necessary to try to minimize the uncertainty that the diagnosis of VUS.

## Conclusions

In conclusion, we found an association between ultrasonic soft markers and genetic abnormalities in fetuses. Presence of ultrasonic soft markers, especially short femur, thickened nuchal translucency, choroid plexus cyst, absent nasal bone, ventriculomegaly, prenatal diagnosis and SNP array are recommended. The results of this study indicate that the genetic etiology of fetuses with ultrasonic soft markers is not only related to chromosomal abnormalities, but also related to abnormal CNVs. SNP array can complement for the deficiency of conventional karyotyping and improve the rate of chromosomal abnormalities. SNP array should be used for fetuses with ultrasonic soft markers and normal conventional karyotyping.

## Data Availability

The microarray data from this study were submitted to the Gene Expression Omnibus repository (https://www.ncbi.nlm.nih.gov/geo/query/acc.cgi?acc=GSE163799).

## Abbreviations

CNVs: Copy number variations
SNP: Single nucleotide polymorphism
VUS: Uncertain clinical significance
